# Plasma-deposited fluoropolymer film mask for local porous silicon formation

**DOI:** 10.1186/1556-276X-7-344

**Published:** 2012-06-26

**Authors:** Thomas Defforge, Marie Capelle, François Tran-Van, Gaël Gautier

**Affiliations:** 1 Université François Rabelais de Tours, GREMAN UMR CNRS 7347, 16 Rue Pierre et Marie Curie, BP 7155, Tours Cedex 2, 37071, France; 2 ST Microelectronics, 16 Rue Pierre et Marie Curie, BP 7155, Tours Cedex 2, 37071, France; 3 Laboratoire de Physico-Chimie des Matériaux et Biomolécules, Université François Rabelais de Tours, EA 6299, Parc de Grandmont, Tours, 37200, France

**Keywords:** Fluoropolymer, Fluorocarbon polymer, Porous silicon localization, Chemical resistance, Inert masking layer

## Abstract

The study of an innovative fluoropolymer masking layer for silicon anodization is proposed. Due to its high chemical resistance to hydrofluoric acid even under anodic bias, this thin film deposited by plasma has allowed the formation of deep porous silicon regions patterned on the silicon wafer. Unlike most of other masks, fluoropolymer removal after electrochemical etching is rapid and does not alter the porous layer. Local porous regions were thus fabricated both in p^+^-type and low-doped n-type silicon substrates.

## Background

Silicon electrochemical etching is known as a low-cost technique to rapidly and easily produce large areas of porous silicon (PoSi). The large range of possible morphologies of PoSi makes it an attractive material for numerous applications. Micro- and mesopores (i.e., with pore diameter < 2 nm and 2 to 50 nm, respectively) are intensively studied in photoluminescence [1], microelectromechanical systems (MEMS) [2], or RF device [3,4] domains, whereas macropores may be employed in through-silicon via (TSV), photonics [5], or membrane [6,7] applications. Despite the wide range of applications and its low production cost, silicon anodization suffers from several issues that limit its industrialization. The localization of porous regions on the silicon wafer is the most restrictive one, especially for microelectronic applications.

The reason of localization issues is directly linked to the fabrication process. The PoSi is produced in hydrofluoric acid (HF)-based solutions, often at high acid concentration. This acid is highly corrosive; thus, only few materials are chemically resistant in this solution for long-duration anodizations since some specific applications require the formation of deep local PoSi regions. Moreover, for some materials, the application of an anodic bias during the PoSi formation may affect their chemical stability.

For most of the above-mentioned applications, the mask must be removed after local anodization. Moreover, the mask removal must not deteriorate the underlying porous layer. Micro- and mesoporous layers are very sensitive to their chemical environment and to the temperature because of their high developed surface. A mask removal technique that also etches or oxidizes the PoSi is thus forbidden. Regarding the macropores, their thick sidewalls and lower developed surface ensure a relative chemical resistance compared to micro/mesoporous layers.

Finally, local formation of PoSi requires a mask compatible with a photolithographic process. It must also be selectively removed through a photoresist or a hard mask.

Masking layers with various characteristics have been studied for two decades. However, no mask lived up to the three above-mentioned conditions. The simplest masking layer that may be considered is the photoresist. Nevertheless, the mask is rapidly removed in HF-based solution [8]. Indeed, only short etching durations may be considered. To increase the masking layer resistance, buffered HF (BHF) may also replace the HF as electrolyte [9]. However, the etching conditions in BHF are completely different from those in HF-based solution because of its higher viscosity.

Silicon dioxide may also be employed as a mask [10]. However, this material presents the same issue as the photoresist: the dissolution rate is very high in HF especially at high concentration. Nevertheless, the oxide layer can be covered by a silicon polycrystalline film deposited by chemical vapor deposition (CVD) [11,12]. The polycrystalline layer protects the silicon from the etching, but the thin SiO_2_ layer border (or edge) is still in contact with HF. The polycrystalline film can thus be removed by lift-off at the edges of the porous region [13].

Silicon carbide (SiC) thin films were also experimented as a masking layer for PoSi localization [8,14,15]. Crystalline SiC is not suitable because it is known to become porous in HF-based solution [16]. However, amorphous SiC films deposited by CVD present semi-insulating properties and thus remain inert during anodization. The main issues of this material are its patterning and removal processes. The SiC is locally removed by CF_4_ plasma etching. This gas is not selective to silicon and could deteriorate the substrate during the patterning or the porous layer during its removal. The problem is the same with silicon nitride (Si_x_N_y_) as a masking layer [15,17]. However, depending on the deposition technique and the Si/N stoichiometric ratio, the mask etch rate in HF solution can be reasonably low [18].

Carbon layers were also studied as a mask [19]. Deposited by electron beam technique, this thin film presents a high resistance in HF depending on the deposition parameters. However, its chemical resistance was only studied for short-time anodization. Moreover, the deposition tool is not widely used even in research laboratories, and it is not suitable for high-throughput production. No post-anodization mask removal was performed, but one can assume that O_2_ plasma may be an efficient film stripping option.

According to the literature, all of these masking layers present some drawbacks or limitations for the silicon electrochemical etching and mask removal.

In the present paper, we propose an innovative fluoropolymer (FP)-based layer that owns high chemical resistance into HF-based electrolyte, even under anodic bias and damage-free removal. This mask was studied in low-doped n-type and highly doped p-type substrates and in several electrolytes to perform deep local PoSi regions.

## Methods

### Patterning procedures

**Figure 1:**
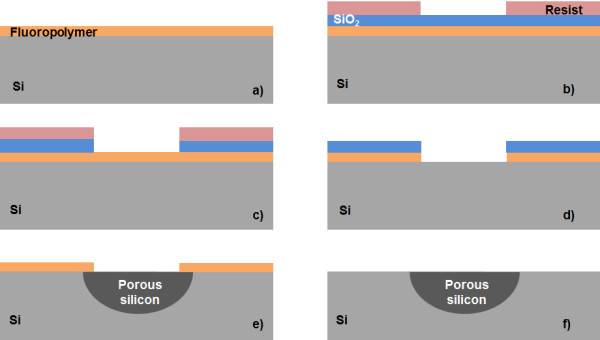
** Typical process used to fabricate the openings in the FP.** (**a**) The FP is plasma-deposited. (**b**) A silicon dioxide layer is deposited by PECVD, and the photoresist is deposited by spin coating. The photoresist is patterned by photolithography. (**c**) The oxide is etched through the resist. (**d**) The FP is then etched through the oxide mask with O_2_ plasma that also removes the remaining resist. (**e**) The sample is anodized to form porous silicon; the oxide is etched during the anodization. (**f**) Finally, the FP is totally removed with O_2_ plasma.

The novel mask was a 150-nm FP deposited in an inductively coupled plasma equipment (*cf.* Figure 1, step a). The exact chemical composition is not yet known, but it is assumed to be a fluorocarbon polymer since the precursor gases are C_2_H_4_ and CHF_3_. An 80-W radiofrequency plasma was applied during the whole deposition process at ambient temperature and under 50-mT pressure. With these conditions, the deposition rate was 35 nm/min.

Since the FP and the resist are both chemically attacked by O_2_ plasma, the patterning of the mask cannot be performed without hard mask. So, once the FP was achieved, silicon dioxide was deposited as hard mask by plasma-enhanced chemical vapor deposition (PECVD) (*cf.* Figure 1, step b). The oxide layer was patterned, thanks to standard photolithography, followed by HF etching (step c). The removal of the photoresist by O_2_ plasma also strips the unmasked FP (step d). The regions masked with the oxide remained intact. During the electrochemical etching, the oxide hard mask is naturally etched in the HF-based electrolyte (step e). After anodization, the FP mask was removed with O_2_ plasma at the ambient without damaging the fabricated PoSi region (Figure 1, step f). Fourier transform infrared spectroscopy analysis was conducted and showed that no oxide was grown at the surface of PoSi during the mask removal. The oxide hard mask can be replaced by other materials such as metal or resist. However, the hard mask deposition before the patterning of FP must be achieved at low temperature (<200°C) to avoid polymer thermal deterioration.

### Anodization conditions

The local electrochemical etching of PoSi was performed in two different types of silicon substrates: a highly doped p-type (111)-oriented wafer and a low-doped n-type (100)-oriented one. It is well known that these kinds of silicon resistivities lead to the formation of very different PoSi morphologies [20]. P^+^-type silicon enables the formation of mesoporous layers whereas a low-doped n-type substrate leads to the development of macropores grown along the [100] direction. In both cases, the anodization was performed through the openings of the FP mask in a double-tank electrochemical cell.

### P^+^-type substrate: mesoporous silicon

**Figure 2:**
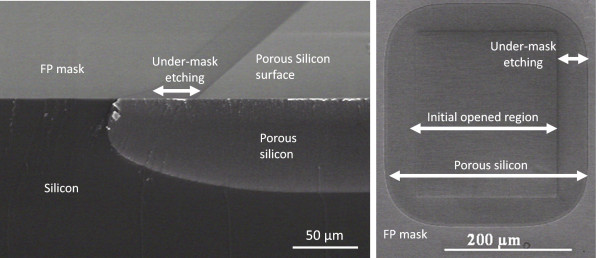
** SEM views of local PoSi regions fabricated in a 20-mΩ cm p-type (111) silicon substrate.** Slice-tilted (on the left) and top (on the right) views of local porous regions were performed by SEM. The anodization was performed at 28 mA cm^−2^ for 45 min in a HF (30 wt.%)-acetic acid (25 wt.%) electrolyte. The depth of the locally produced PoSi region is 80 μm. A 60-μm under-mask etching was measured.

The selective fabrication of PoSi in a highly doped silicon substrate is a real challenge for the integration of radiofrequency circuits. Thus, it allows the integration of both active and passive devices on the same wafer by local porous region formation. The aim is to take advantage of the insulating properties of the PoSi (as compared to bulk silicon) to improve the performances of passive devices, such as filters [3,4]. That is why local PoSi regions were fabricated in a 20-mΩ cm p-type silicon substrate according to the conditions described above (Figure 2).

Mesoporous layers were fabricated by electrochemical etching of a highly doped (*ρ* = 20 mΩ cm) p-type (111) silicon wafer. The electrolyte was composed of HF (30 wt.%), acetic acid (25 wt.%), and water. A constant current density of 28 mA cm^−2^ was applied during the whole anodization. Anodizations were performed up to 4 h. With these conditions, an average porosity of 50% was obtained (estimated by weight measurements).

### N-type substrate: ordered macropore arrays

Since the electrochemical etching was performed in a double-tank electrochemical cell, the semiconductor backside is polarized by the electrolyte [21]. To ensure an efficient ohmic contact in low-doped n-type substrates (*ρ* = 26 to 33 Ω cm), an n^+^ layer was performed on the backside of the samples by dopant diffusion or implantation. To achieve ordered macropores, initiation micro-pyramids were etched in the frontside (side of PoSi formation) through the FP mask. It is important to notice that the FP film is also highly resistant to concentrated (20 wt.%) alkaline solution (KOH) even at high temperature (80°C) during the development of the micro-pyramids. The samples were immersed into a HF (2.4 wt.%)-cetyltrimethylammonium chloride (CTAC, 120 ppm)-water mixture. CTAC was employed as a surfactant to ensure efficient electrolyte penetration into the porous media and hydrogen removal during the electrochemical etching. Triton X-100® (provided by Fisher Scientific®, Illkirch, France) was also studied as a wetting agent. The anodization was performed under potentiostatic control. Since PoSi formation is driven by hole concentration in the semiconductor, a halogen lamp backside illumination was essential for carrier generation in this substrate during the whole electrochemical etching process. Once the macropores were etched, the samples were rinsed and dried. The FP was then removed by O_2_ plasma.

## Results and discussions

After the anodization of the p-type substrate, the FP adheres well to the silicon surface, even on the borders of the openings and for long-duration anodizations. Nevertheless, scattered small defects were noticed after the electrochemical etching on the surface of the thin FP layer (150 nm). Since they were only observed with highly doped silicon substrates and at high voltage (above 7 V), it can be assumed that the defects are produced by an electrical breakdown of the FP. The defects could be decreased by raising the FP thickness or by increasing its density by film annealing.

**Figure 3:**
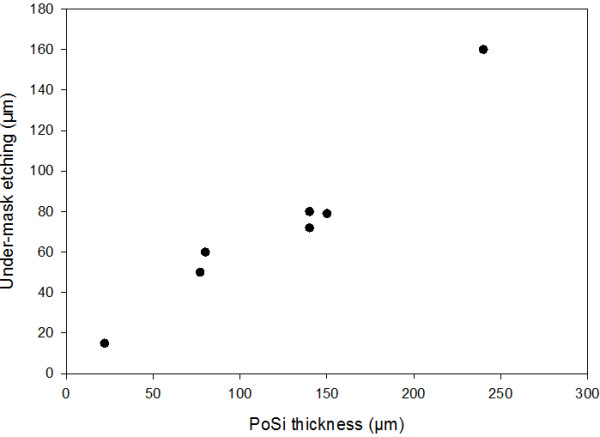
** Under-mask etching as a function of the PoSi region thickness.** The anodizations were performed at 28 mA cm^−2^ in a HF (30 wt.%)-acetic acid (25 wt.%) electrolyte in p-type 20-mΩ cm silicon.

Figure 2 exhibits the quasi-isotropic etching behavior in this material since PoSi is fabricated under the mask. For a given anodic current density, the under-mask etching seems to be proportional to the PoSi thickness (Figure 3). Ratios from 0.5 to 0.7 were obtained between the under-mask etching length and the PoSi thickness.

**Figure 4:**
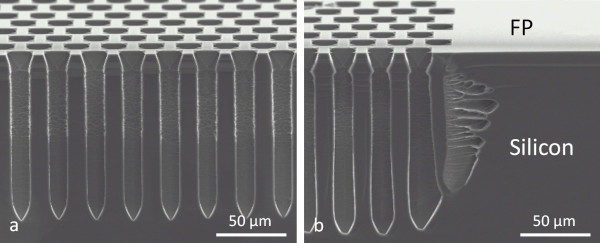
** High-aspect-ratio local macropore etching in low-doped n-type silicon.** Macropores of 15.5 μm in diameter with 30-μm pitch were etched into HF (2.4 wt.%)-CTAC (120 ppm) for 4 h. The electrochemical etching was performed under potentiostatic control (3.25 V/CE) and 130-W backside illumination. (**a**) Middle of the macropore array region. (**b**) Border of the macropore array region.

**Figure 5:**
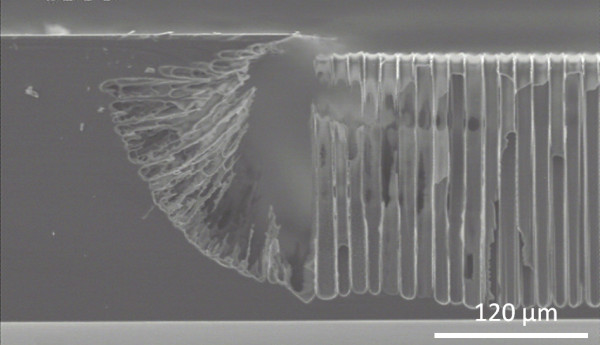
** Over-etching at the edges of the mask during local macropore formation.** The electrochemical etching was performed in low-doped n-type silicon (30 Ω cm) under potentiostatic control (4 V/CE) for 3 h under 130-W backside illumination. The electrolyte was composed of HF (2.4 wt.%) and CTAC (120 ppm).

In the low-doped n-type substrate, ordered macropores were grown to perform local macropore arrays for TSV applications. In this case, the macropores were etched for several hours (depending on the required pore depth). Unlike the p^+^-type substrate, no default on the FP surface was observed after the anodization (*cf.* Figure 4). This behavior could be imputed to the protection of the mask by a thick (few microns) space charge region (SCR) in the underlying semiconductor. Nevertheless, the borders of the masking regions exhibit a slight over-etching. This undercut is linked to the photo-generation of carriers on the whole backside surface. The concentration of holes available at the edge is thus much higher than that in the middle of the opened region. This often leads to highly porous borders and sometimes completely eroded regions, especially at high applied current density (*cf.* Figure 5). Moreover, the SCR that protects the sidewalls from dissolution is not thick enough to protect the borders. A complete study of under-mask etching around the edges of macropore arrays in a low-doped n-type substrate was performed by Tao and Esashi [15,22]. Due to its observations, the under-mask etching could be limited, thanks to backside illumination localization. Chromium/gold (Cr/Au) is a mask commonly deposited on the backside to limit the photo-generation to desired regions. Thus, the edges are under-etched (in this case, pore dying is observed) instead of eroded [17].

**Figure 6:**
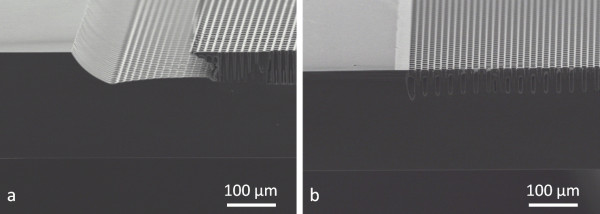
** Comparison of electrochemical parameters regarding the over-etching at the porous region edges.** In both cases, a 15-μm-pitch, 30-Ω cm n-type substrate was etched for 3 h into a HF (2.4 wt.%)-Triton X-100® (500 ppm) mixture. (**a**) The electrochemical etching was performed under 6-V bias (vs. CE) and 50-W backside illumination. (**b**) The macropores were etched under 3.5 V/CE under 140-W backside illumination.

During the experiments, it has been found that the applied current density and/or potential is essential to limit the edge defects. Figure 6 compares two 15-μm pitch macropore arrays etched under potentiostatic control. The first one, at high potential (6 V/counter electrode (CE)) and low illumination power (50 W), leads to a large eroded region at the edge (approximately 150 μm) as illustrated in Figure 6a, whereas the second one etched at low potential (3 V/CE) and high illumination power (140 W) leads to almost perfect borders (*cf.* Figure 6b). This behavior is imputed to the poor photo-generation in the first case (Figure 6)a. The photocurrent is sufficient in the middle patterned region to ensure stable pore growth (thanks to high voltage). However, it is too high at the edges where the photocurrent is assumed to be higher; thus, the porosity increases and erosion is observed.

Different pattern densities were also analyzed in the same substrate: the highest is the pore density; the worst are the edges to ensure a stable pore growth in the middle of the opened area. The higher current density (respectively, bias) that must be provided to ensure a stable pore growth is assumed to be responsible for this behavior. To increase the pattern density, the objective is to decrease the substrate resistivity to reduce the stable growth current density [23].

## Conclusions

The use of fluoropolymer film to protect silicon regions from anodization in HF electrolyte was investigated. This film was studied for two different substrates (p^+^-type and n-type Si) under anodic bias: no porous silicon was observed under the mask (except for the border over-etching that cannot be avoided) since the polymer is inert in HF-based solution. Only few defects at the surface of the FP were observed at high voltages in the case of p^+^ substrates only. However, these defects can be reduced by optimizing the electrical properties of the mask (currently under investigation). This mask presents many advantages compared to others, especially its fast and non-altering removal after electrochemical etching. It is also compatible with the common microelectronic industry processes. This type of masking layer may thus be employed for numerous microelectronic applications such as MEMS, Si/PoSi hybrid substrates, or 3D integration. Thanks to its chemical resistance, the FP may also be employed as a protection for the underlying layers for a back-end electrochemical etching process. Physical and electrical characterizations of this mask are currently under investigations.

## Abbreviations

BHF, buffered HF; CE, counter electrode; CTAC, cetyltrimethylammonium chloride; CVD, chemical vapor deposition; FP, fluoropolymer; HF, hydrofluoric acid; PECVD, plasma-enhanced chemical vapor deposition; SCR, space charge region; SiC, silicon carbide; SixNy, silicon nitride; PoSi, porous silicon; TSV, through silicon via.

## Competing interests

The authors declare that they have no competing interests.

## Authors’ contributions

TD and MC wrote the manuscript. TD and MC developed the process of FP as mask for silicon anodization in n- and p-type silicon. FT-V and GG participated in the conception of the study and revised the manuscript for important intellectual contents. All authors read and approved the final manuscript.

## References

[B1] Cullis A Canham L Calcott P The structural and luminescence properties of porous silicon J Appl Phys 1997 82 909 10.1063/1.366536

[B2] Lang W Silicon microstructuring technology Mat Sci & Eng R: Reports 1996 17 1 55 10.1016/0927-796X(96)00190-8

[B3] Billoué J Gautier G Ventura L Integration of RF inductors and filters on mesoporous silicon isolation layers Phys Status Solidi (a) 2011 208 1449 1452 10.1002/pssa.201000027

[B4] Capelle M Billoué J Poveda P Gautier G N-type porous silicon substrates for integrated RF inductors IEEE Trans Electron Dev 2011 58 4111 4114

[B5] Schilling J Müller F Matthias S Wehrspohn R Gösele U Busch K Three-dimensional photonic crystals based on macroporous silicon with modulated pore diameter Appl Phys Lett 2001 78 1180 10.1063/1.1351533

[B6] Desplobain S Gautier G Ventura L Bouillon P Macroporous silicon hydrogen diffusion layers for micro-fuel cells Phys Status Solidi (a) 2009 206 1282 1285 10.1002/pssa.200881081

[B7] Matthias S Müller F Asymmetric pores in a silicon membrane acting as massively parallel brownian ratchets Nature 2003 424 53 57 10.1038/nature01736 12840755

[B8] Steiner P Lang W Micromachining applications of porous silicon Thin Solid Films 1995 255 52 58 10.1016/0040-6090(95)91137-B

[B9] Kleimann P Badel X Linnros J Toward the formation of three-dimensional nanostructures by electrochemical etching of silicon Appl Phys Lett 2005 86 183108 10.1063/1.1924883

[B10] Nassiopoulos A Grigoropoulos S Canham L Halimaoui A Berbezier I Gogolides E Papadimitriou D Sub-micrometre luminescent porous silicon structures using lithographically patterned substrates Thin Solid Films 1995 255 329 333 10.1016/0040-6090(94)05675-4

[B11] Kaltsas G Nassiopoulou A Frontside bulk silicon micromachining using porous-silicon technology Sens Actuators A 1998 65 175 179 10.1016/S0924-4247(97)01669-5

[B12] Gautier G Kouassi S Desplobain S Ventura L Macroporous silicon hydrogen diffusion layers for micro-fuel cells: from planar to 3D structures Microelectron Eng 2012 90 79 82

[B13] Sancho A Arizti F Gracia F Porous silicon for the development of capacitive microstructures Microelectron Eng 2009 86 2144 2148 10.1016/j.mee.2009.02.031

[B14] Wang H Welker B Gao Y Federici J Levy R Photolithographic patterning of porous silicon using silicon nitride and silicon carbide masks Mat Lett 1995 23 209 214 10.1016/0167-577X(95)00050-X

[B15] Tao Y Esashi M Local formation of macroporous silicon through a mask J Micromech Microeng 2004 14 1411 10.1088/0960-1317/14/10/017

[B16] Shor J Kurtz A Photoelectrochemical etching of 6 H-SiC J Electrochem Soc 1994 141 778 10.1149/1.2054810

[B17] Homma T Sato H Mori K Osaka T Shoji S Area-selective formation of macropore array by anisotropic electrochemical etching on an n-Si (100) surface in aqueous HF solution J Phys Chem B 2005 109 5724 5727 16851620 10.1021/jp045822n

[B18] Izuo S Ohji H French P Tsutsumi K A novel electrochemical etching technique for n-type silicon Sens Actuators A 2002 97 720 724

[B19] Djenizian T Santinacci L Hildebrand H Schmuki P Electron beam induced carbon deposition used as a negative resist for selective porous silicon formation Surf Sci 2003 524 40 48 10.1016/S0039-6028(02)02545-1

[B20] Lehmann V Stengl R Luigart A On the morphology and the electrochemical formation mechanism of mesoporous silicon Mat Sci Eng B 2000 69 11 22

[B21] Lang W Steiner P Sandmaier H Porous silicon: a novel material for microsystems Sens Actuators A 1995 51 31 36 10.1016/0924-4247(95)01066-1

[B22] Tao Y Esashi M Macroporous silicon-based deep anisotropic etching J Micromech Microeng 2005 15 764 10.1088/0960-1317/15/4/013

[B23] Barillaro G Strambini L Controlling macropore formation in patterned n-type silicon: existence of a pitch-dependent etching current density lower bound Electrochem Comm 2010 12 1314 1317 10.1016/j.elecom.2010.07.008

